# 

**Published:** 1812-07

**Authors:** 


### MEDICAL AND PHYSICAL

### JOURNAL.

CONDUCTED BY

SAMUEL FOTHERGILL, M.D.

AND

WILLIAM ROYSTON, ESQ. F.L.S.

VOL. XXVIII.

FROM JULY TO DECEMBER, 1812.

Et quoniam variant morbi, variabimus artes;

Mille mali species, mille salutis erunt.

*LONDON:*

PRINTED FOR RICHARD PHILLIPS; BY J. ADLARD, 23, BARTHOLOMEW-CLOSE, AND 39, DUKE-STREET, SMITHFIELD; AND SOLD BY J. SOUTER, NO. 1, PATERNOSTER-ROW.

### MONTHLY CATALOGUE OF MEDICAL BOOKS.

TRANSACTIONS of a Society for the Improvement of Medical and Chirurgical Knowledge. Illustrated with copper-plates. Vol. 3. 8vo.—Nichol.

Forster (Thomas), Physiological Reflections on the destructive Operation of Spirituous and Fermented Liquors on the Animal System. 8vo.—Underwood.

Leese (Edward), Explanation of the Causes why Vaccination has sometimes failed to prevent Small-pox; and also a Description of a Method, confirmed by Experience, of obviating such Causes. 8vo.—Underwood.

Observations on the Nature and Treatment of Tinea Capitis, or Scald Head; and on Impaired Vision, arising from Opacity of the Cornea. By Thomas Luxmoore.—Highley.

Barclay (Dr. John), Description of the Arteries, with copious Notes. 12mo.—Bryce and Co.

The Tenth Edition of Mr. Chamberlaine's Treatise on the Dolichos Pruriens, is just published, and on sale at the different medical booksellers.

---

### *NOTICES TO CORRESPONDENTS.*

*We thank the following Gentlemen for their obliging Communications: James Little, Surgeon of his Majesty's Ship Salvador del Mundo; Dr. Clarke; A. Fogo; Medicus; Dr. S. M.—T. E. B. &c. &c.*

*The Meteorological Table, for this Month, is unavoidably omitted, from the Indisposition of the Gentleman who usually prepares it.*

*The Editors decline inserting the Letter signed* PHILANTHROPHIA; *the Motive of the Writer may be pure, but the Subject is disgusting.*

